# Adoption of Innovations for Cacao Pest and Disease Management in Huila, Colombia: Insights From Regression and Social Network Analysis

**DOI:** 10.1155/sci5/3375961

**Published:** 2026-10-26

**Authors:** Sonia Mercedes Polo-Murcia, Diego Alberto Navarro-Niño, César Augusto Terán-Chaves, Eleonora Rodríguez-Polanco, Bayardo Parra-Alferes, Jhon Jairo Zuluaga-Peláez

**Affiliations:** ^1^ Universitat Politécnica de Valencia 46022 Valencia Spain upv.es; ^2^ Grupo de Investigación Agricultura Inteligente Para la Sostenibilidad de los Sistemas de Producción en el Trópico Universidad Nacional de Colombia Bogotá Colombia unal.edu.co; ^3^ Centro de Investigación Nataima Corporación Colombiana de Investigación Agropecuaria—AGROSAVIA km 9 Vía Chicoral-Espinal Espinal 733529 Colombia

**Keywords:** beta regression, fractional regression, innovation adoption, *Moniliophthora roreri*, rural extension, social networks, sustainability, technical efficiency, *Theobroma cacao* L.

## Abstract

Cacao (*Theobroma cacao* L.) is fundamental to rural economies in Colombia, with Huila contributing 8% of the national output. However, its production is severely constrained by pests and diseases, such as *Moniliophthora roreri*, *Phytophthora palmivora*, and *Carmenta foraseminis*, which can cause yield losses of 30%–100%. Limited adoption of integrated pest and disease management (IPDM) practices among smallholders exacerbates these constraints, undermining sustainability. This study identifies the socioeconomic and institutional factors associated with IPDM adoption in Huila. Because the data are cross‐sectional, relationships are interpreted as associations rather than causal effects. A survey of 156 cacao farmers across six municipalities (2024) was analyzed using beta and fractional regression models, suitable for proportional outcomes, complemented by co‐occurrence and social network analysis. The Innovation Adoption Index (INAI), rebuilt to exclude items used as predictors, averaged 0.63, indicating moderate adoption. After controlling for farmer, farm, and institutional characteristics, participation in training (average marginal effect, AME = 0.148; *p* = 0.024), access to extension services (AME = 0.116; *p* = 0.004), and credit use (AME = 0.082; *p* = 0.007) were positively associated with adoption, whereas total farm area was negatively associated (AME = −0.005; *p* = 0.004). Association membership was not individually significant, although the institutional block was jointly significant (*p* < 0.001), suggesting an indirect channel operating through training and extension. Social network analysis identified extension agents as central knowledge brokers but revealed weak farmer‐to‐farmer ties. Strengthening cooperative networks, participatory learning, and targeted extension could enhance IPDM diffusion and reinforce sustainable cacao production in Colombia.

## 1. Introduction

*Theobroma cacao* L., a perennial crop native to the Amazon region and belonging to the Malvaceae family, represents a cornerstone of sustainable agriculture, thanks to its significant economic and cultural importance and remarkable genetic diversity [1]. Globally, cacao cultivation underpins the livelihoods of millions of smallholder farmers. This importance is magnified by the rising demand for cacao products, driven by a burgeoning middle class in Asia and Africa. Leading producers, such as Côte d’Ivoire and Ghana, dominate the global supply, while Latin American nations, such as Ecuador, Brazil, and Colombia, are pivotal contributors to the premium segment of the market. Colombia, in particular, is recognized internationally for its fine and aromatic cacao, which commands higher prices in specialty markets [2, 3].

In Colombia, cacao is not only an economic commodity but also a social catalyst, integral to rural development and postconflict policies aimed at replacing illicit crops. As of 2023, Colombia had 256,325 ha under cacao cultivation, producing 140,109 metric tons annually, representing a 3.1% increase from the previous year. However, the average yield of 0.6 tons per hectare reflects persistent productivity constraints. The Department of Huila, an emerging cacao‐producing region, cultivated 8534 ha with an average yield of 0.62 tons per hectare. Beyond its economic potential, the sector directly supports over 65,000 families across 30 departments, cementing its role in rural livelihoods [4, 5].

Despite this progress, the cacao sector in Huila faces significant challenges that hinder its potential to achieve sustainable growth. Chief among these are biotic stresses caused by pests and diseases, such as *Moniliophthora roreri* (frosty pod rot), *Phytophthora palmivora* (black pod disease), and *Carmenta foraseminis* (cacao fruit borer). These challenges result in severe yield losses, ranging from 30% to 100%, depending on regional conditions and management practices. For example, *M. roreri* is highly aggressive and adaptable, capable of devastating entire plantations due to its rapid reproductive cycle and efficient spore dispersion [6, 7]. *P. palmivora* adds to these losses by attacking multiple organs of the cacao plant, while *C. foraseminis* inflicts direct fruit damage, with infestation rates reaching up to 77% in some regions [8, 9]. For smallholder farmers in Huila, who rely on limited financial and technical resources, these threats exacerbate their vulnerability, often restricting them to reactive measures that fall short of sustainable solutions.

Agricultural innovations provide a pathway to mitigate these challenges and enhance the sustainability of cacao production. Defined as the implementation of new technologies, practices, and strategies to improve productivity and resilience, agricultural innovation has been widely studied for its role in driving rural development [10, 11]. Metrics, such as the Innovation Adoption Index (INAI) and the Rate of Adoption of Innovations (TAI), are instrumental in assessing the diffusion of these practices among farmers [12, 13]. Moreover, social networks play a critical role in this diffusion process. Farmers occupying central positions within knowledge‐sharing networks often act as opinion leaders, accelerating the adoption of technologies among their peers [14, 15]. This highlights the importance of cooperative associations, extension services, and training programs in facilitating the transfer of knowledge and promoting innovation adoption.

Smallholder cacao farmers in Huila operate within a challenging socioeconomic and environmental context. The average farm size is typically less than 5 ha, with cacao production often integrated into diversified subsistence farming systems. Farmers face significant barriers, including limited access to credit, low levels of formal education, and inadequate technical support. These constraints hinder the adoption of advanced management practices and perpetuate reliance on traditional methods that are insufficient to address persistent biotic stresses [16, 17]. However, the strong community ties and active participation in farmer associations in Huila present an opportunity for leveraging social networks to drive innovation.

This study examines the factors associated with the adoption of innovations for managing *M. roreri*, *P. palmivora*, and *C. foraseminis* in smallholder cacao farming in Huila, Colombia. Because the study relies on cross‐sectional data, we frame our expectations as associations rather than causal effects. We expected that: (1) Access to training, extension services, and credit would be positively associated with adoption, reflecting the role of knowledge and financial resources in the uptake of integrated pest and disease management (IPDM) strategies; (2) a more central position within knowledge‐sharing networks would be associated with higher adoption, consistent with the role of well‐connected farmers in technology diffusion; and (3) larger farm size would be negatively associated with adoption, as the coordinated implementation of labor‐intensive practices becomes more demanding over larger areas. These expectations align with previous research on agricultural innovation diffusion, emphasizing the role of socioeconomic and network‐based factors in shaping farmer decision‐making [18, 19].

To empirically assess these relationships, we employ beta and fractional regression models, well‐suited for analyzing bounded adoption indices. Additionally, social network analysis (SNA) is integrated to explore the structure and influence of farmer interactions on innovation diffusion. Although the determinants of agricultural technology adoption have been widely studied, and social networks are increasingly recognized as channels of knowledge diffusion in smallholder systems [20, 21], few studies have jointly examined the socioeconomic determinants of adoption and the structure of knowledge exchange networks within the same cacao‐producing population, and fewer still have done so for IPDM in Colombian smallholder systems, where most prior work treats the network dimension descriptively or separately from the econometric analysis of adoption. By combining econometric modeling with network analysis, this study addresses that gap and offers a comprehensive perspective on the drivers and barriers to IPDM adoption in a low‐resource setting. The findings contribute to evidence‐based policymaking and extension strategies aimed at strengthening cooperative networks, enhancing participatory learning models, and addressing technology adoption gaps in Huila’s cacao sector. Furthermore, by situating this research within a broader regional and global context, we provide valuable insights for smallholder innovation adoption in tropical agriculture, with implications for similar production systems worldwide [22].

## 2. Materials and Methods

### 2.1. Study Area and Productive System

The study was conducted in 2024 in the Department of Huila, located in the southern region of the Colombian Andes. Geographically, Huila spans latitudes 01°55′45″ to 03°48′51″ N and longitudes 74°23′51″ to 76°37′11″ W. Huila exhibits substantial environmental heterogeneity associated with its altitudinal gradient, ranging from the Magdalena River valley to high Andean and páramo ecosystems. Annual precipitation varies across the department from approximately 900–1000 mm in the Magdalena River valley to more than 1500 mm in higher elevation areas, while intermediate zones generally receive between 1100 and 1500 mm·yr^−1^. According to the land‐suitability assessment conducted by the Colombian Rural Agricultural Planning Unit, approximately 207,325 ha (11.4% of the departmental area) are suitable for commercial cacao cultivation in Huila, of which 118,756 ha are classified as highly suitable, 55,131 ha as moderately suitable, and 33,439 ha as marginally suitable [23]. Most cacao‐growing areas belong to the dry inter‐Andean valley production zone, generally below 900 m a.s.l., characterized by warm temperatures (26°C–30°C), predominantly loamy soils, and annual precipitation generally below 1500 mm—conditions under which supplemental irrigation may be required to avoid water deficits [24]. To enhance resilience to climatic variability and maintain ecosystem functions, cacao is commonly cultivated within agroforestry systems, where shade trees regulate microclimatic conditions, protect the soil, conserve biodiversity, and provide multiple ecosystem services [25].

The study focused on six municipalities—Rivera, Gigante, Campoalegre, Algeciras, Hobo, and Yaguará—selected on three converging criteria. First, they are among the principal cacao‐producing municipalities of Huila: According to the official municipal agricultural evaluations, in 2024 Rivera, Gigante, Campoalegre, and Algeciras ranked first, fourth, fifth, and sixth in the department by planted area (919, 545, 506, and 434 ha, respectively), and the six municipalities together accounted for approximately 2586 ha—close to one‐third (≈32%) of the department’s planted cacao area and ≈29% of its production [5]. Second, four of them (Campoalegre, Gigante, Rivera, and Algeciras) constitute the intervention area of the regional research and development project that framed this study (BPIN 2021000100166), which ensured institutional access to producers and to technical records. Third, the set spans the main agroecological gradient under which cacao is grown in the department, from the warmer, drier inter‐Andean valleys to slightly more humid settings, providing a representative range of production conditions (Figure 1). Hobo and Yaguará were deliberately retained to broaden this agroecological coverage; as they are comparatively minor producers (115 and 66 ha in 2024, respectively), they contributed correspondingly small samples and, as detailed in Section 2.2, their results are interpreted descriptively and with caution.

**FIGURE 1:**
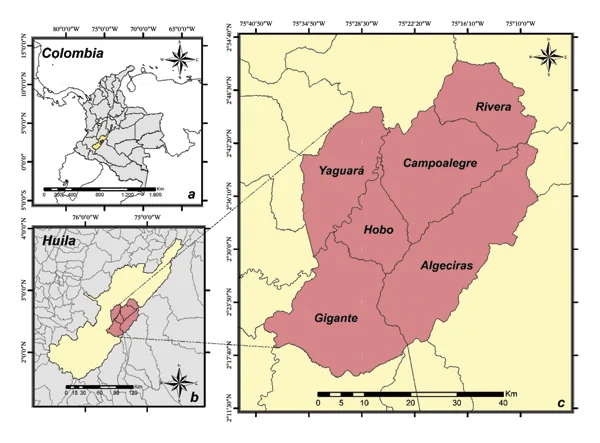
Study area. (a) Map of Colombia highlighting the Department of Huila in light yellow. (b) Enlarged map of Huila showing the specific locations of the selected municipalities for the study—Gigante, Algeciras, Campoalegre, Rivera, Hobo, and Yaguará (marked in coral red). (c) Detailed map of the selected municipalities, with cacao cultivation areas (*Theobroma cacao L.*) represented in coral red.

Despite these favorable agroecological conditions, cacao production in Huila faces significant threats from pests and diseases, including frosty pod rot (*M. roreri*), black pod rot (*P. palmivora*), and the cacao fruit borer (*C. foraseminis*). *M. roreri* is a major cause of productivity losses, with reported yield losses of 30%–100%, severely affecting cacao pods through its high reproductive efficiency and adaptability. Similarly, *P. palmivora* damages not only the pods but also the trunks of cacao trees, causing substantial economic losses, while *C. foraseminis*, a lepidopteran pest, physically damages fruits and seeds, with larvae boring into pods and compounding the challenges faced by producers. The visual symptoms caused by these pathogens and pests on cacao fruits are illustrated in Figure 2, where the characteristic damage by *M. roreri* (a), *P. palmivora* (b), and *C. foraseminis* (c) is shown.

**FIGURE 2:**
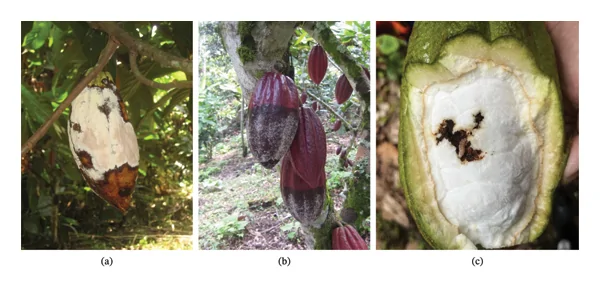
Characteristic symptoms of the main cacao pod pests and diseases in Huila. (a) Frosty pod rot caused by *Moniliophthora roreri*, showing external necrotic lesions and white sporulation on the pod surface; (b) black pod rot caused by *Phytophthora palmivora*, with dark, firm lesions that spread across the pod; and (c) damage by the cacao fruit borer *Carmenta foraseminis*, showing larval entry holes and internal boring of fruits and seeds. Photographs taken in the study area, 2024.

To address these biotic stressors, IPDM is widely adopted in the region. This approach combines cultural practices, such as pruning and the removal of infected pods, with the application of biological and low‐impact chemical products. Additionally, the use of disease‐resistant cacao clones has proven to be an effective strategy for reducing losses and increasing plantation resilience. These measures not only help minimize the impact of pests and diseases but also align with the goals of sustainable agricultural production.

Cacao production in Huila holds considerable socioeconomic importance, involving predominantly smallholder farmers who rely on this crop for their livelihoods. The effective management of the productive system, combined with the adoption of sustainable practices, is crucial for improving the competitiveness and long‐term sustainability of cacao farming in the region. By addressing both agronomic challenges and socioeconomic needs, the cacao sector in Huila can enhance its resilience to environmental and market pressures, ensuring a stable livelihood for its producers.

Although cacao in the region is typically grown under agroforestry arrangements, the composition and density of shade trees were not systematically recorded in this survey and are therefore not quantified here; characterizing shade‐canopy structure and its association with IPDM adoption remains a useful direction for future work.

### 2.2. Data Collection

Data were collected between January and July 2024 through structured face‐to‐face surveys administered to cacao farmers in six municipalities of Huila, Colombia: Gigante, Algeciras, Campoalegre, Rivera, Hobo, and Yaguará.

Because no complete registry of cacao growers was available for these municipalities, a nonprobabilistic snowball (chain‐referral) sampling approach was adopted [26]. Recruitment started with multiple independent seed farmers identified through local producer associations and AGROSAVIA extension networks. Each participant was asked to refer other cacao growers, generating successive recruitment waves that continued until no additional eligible farmers could be identified. Using several institutionally independent seed farmers reduced reliance on a single referral chain and increased coverage of the local farming network.

A total of 159 farmers were interviewed. Three questionnaires were excluded because of missing information on key variables, resulting in a final sample of 156 farmers. The sample was distributed across municipalities as follows: Gigante (*n* = 41), Algeciras (*n* = 40), Campoalegre (*n* = 38), Rivera (*n* = 27), Hobo (*n* = 6), and Yaguará (*n* = 4). Refusal rates were not systematically recorded, although refusals were uncommon because farmers were approached through trusted local contacts.

As with any chain‐referral sampling strategy, the sample may over‐represent farmers who are more closely connected to producer organizations or extension services. Consequently, the adoption rates reported in this study should be interpreted as descriptive of the surveyed farmer network rather than as unbiased estimates of the entire cacao‐growing population of Huila. This limitation mainly affects the external validity of prevalence estimates, whereas the conditional associations examined in the regression models remain informative for the sampled population [27]. Therefore, the study focuses on associations between adoption behavior and explanatory factors rather than on population‐level prevalence estimates.

The survey instrument consisted of two sections. The first collected information on farmer and farm characteristics, including age, educational attainment, farm size, and access to technical assistance. The second focused on the adoption of IPDM practices for controlling *M. roreri* (frosty pod rot), *P. palmivora* (black pod disease), and *C. foraseminis* (cacao fruit borer).

Farmers were asked whether they had implemented each of 21 recommended IPDM practices. Responses were recorded as binary variables, coded as adopted (1) or not adopted (0). The resulting adoption matrix was used to analyze co‐occurrence patterns among practices, identify complementary management strategies, and detect potential gaps in pest and disease management.

### 2.3. Social Networks and Cooperation

To evaluate the role of social networks in innovation adoption, farmers were asked: “From whom have you obtained information or knowledge to implement the practices, technologies, and innovations in your cacao production?” The responses were analyzed using UCINET and Gephi, software tools specialized in SNA [28, 29]. These tools allowed for the identification of interaction patterns and the flow of technical knowledge among network actors. Key social network metrics were calculated:

Degree centrality measures the number of direct ties a farmer has, including incoming ties (in‐degree) and outgoing ties (out‐degree). In‐degree reflects the number of information sources consulted by a farmer, while out‐degree indicates the farmer’s role as a knowledge disseminator [30]. Additionally, the study assessed participation in producer associations, training programs, and knowledge exchanges as determinants of innovation adoption rates.

### 2.4. Calculation of the INAI

The study employed the INAI to quantify the extent of innovation uptake among farmers, as proposed by Aguilar‐Ávila et al. [10]. This index measures the proportion of technological innovations adopted to enhance cacao production systems (Table 1). The INAI is calculated using the following equation [10]:

(1) INAIik∑j=1nInnovjkn,

where *I* *N* *A* *I*_*i* *k*_ INAI for the i‐th farmer in the *k*th category; *I* *n* *n* *o* *v*_*j* *k*_ presence (1) or absence (0) of the *j*th innovation in the *k*th category; *n* is the total number of innovations in the *k*th category.

**TABLE 1:** Criteria and practices for the development of the Innovation Adoption Index (INAI) in cacao disease management.

Code	Description	Rationale for practice selection
S01	Monitors pests and diseases for management	Monitoring is essential for the early detection of pests and diseases, enabling timely interventions. According to Cabrera et al. [31] and Akrofi et al. [32], early detection is critical for developing effective management strategies.
S02	Uses homemade traps for pest management	Homemade traps are cost‐effective and easy to construct. Hagnell et al. [33] report that homemade traps can be as effective as commercial ones for certain pests.
S03	Uses commercial traps for pest management	Commercial traps are often more effective but depend on market availability. Urquhart and Scott [34] found that commercial traps can capture more individuals compared to homemade traps, depending on the pest species.
S04	Identifies Monilia in its different stages of development and its damage	Early identification of Monilia is crucial to prevent fungal sporulation and reduce disease incidence [6].
S05	Identifies the fruit and seed borer of cacao (*Carmenta foraseminis*) and its damage	Effective management of *C. foraseminis* requires recognizing the pest, understanding its biology, and implementing early monitoring programs (Carabalí‐Muñoz et al. [8]).
S06	Performs weekly removal of pods affected by pests and diseases (sanitary rounds)	Weekly removal of diseased pods prevents the formation of fungal spores, which can remain active for up to 3 years [6].
S07	Covers affected fruits with leaf litter	Covering or burying affected fruits reduces pest populations. Carabalí‐Muñoz et al. [8] emphasize that leaving damaged fruits exposed allows pests to complete their life cycle.
S08	Implements solarization practices for pest management	Solarization is a potential tool for pathogen control, but its effectiveness depends on accurate predictive models for treatment duration and substrate depth [35].
S09	Uses pits to dispose of fruits affected by diseases	Disposal pits are effective when combined with practices, such as liming. The thickness of the soil layer is also a critical factor.
S10	Uses lime or dehydrating agents for harvest residues	Lime improves pH and nutrient content during composting but lacks direct antifungal or insecticidal effects [36].
S11	Uses biological products for pest control	Biological control reduces pest populations while minimizing environmental impact. Zelaya‐Molina et al. [37] highlight the importance of eco‐friendly pest control methods.
S12	Uses biological products for disease control	Biological control involves using microorganisms to suppress pathogens. However, its success depends on integrating it into sustainable farming systems [38].
S13	Carries out weed control and management	Weed management is important, but its direct impact on disease control is unclear. However, it can influence pest dynamics [39].
E20	Performs formation pruning on the cacao tree	Formation pruning shapes tree structure but has a secondary impact on pest management compared to other practices.
E21	Performs maintenance pruning on the cacao tree	Maintenance pruning improves tree health by enhancing air circulation and light exposure, which can prevent diseases.
E22	Prunes shade trees in crops associated with forest or fruit trees	Pruning shade trees can be beneficial, but its direct impact on pest and disease management is limited compared to primary practices.
E23	Keeps cacao trees at a maximum height of 3.5 m	Maintaining trees at an optimal height improves accessibility for management and harvesting, facilitating pest control [40].
A25	Has an activity calendar	An activity calendar is crucial for planning and executing agricultural practices efficiently. It enhances time and resource management [41].
A26	Records tasks performed on the crop (date, inputs, and practices)	Recording tasks allows for performance tracking and informed decision‐making. This practice helps evaluate the effectiveness of applied techniques [41].
R33	Identifies champion trees (yield, resistance) in the plantation	Identifying high‐performing trees allows for the selection of disease‐resistant genetic material, contributing to more effective management [24, 42].
R34	Renews and rehabilitates aged cacao plantations	Renewing and rehabilitating aged plantations is essential for maintaining productivity and reducing susceptibility to pests and diseases [40, 43].

*Note:* S: Sanitation (Practices related to the prevention, monitoring, and control of pests and diseases); E: Establishment and Plantation Management (Practices related to planting, maintenance, and physical management of the plantation); A: Administration (Practices related to planning, record‐keeping, and organization of farm activities); R: Reproduction and Genetic Improvement (Practices related to the selection, renewal, and improvement of genetic material).

Key innovations and practices were categorized into dimensions, such as sanitation (S), plantation establishment and management (E), administration (A), and genetic improvement (*R*). These dimensions were identified and validated through expert workshops and aligned with best practices for cacao disease management, particularly targeting *M. roreri*, *P. palmivora*, and *C. foraseminis* (Table 1).

The INAI was conceptualized as a *formative* composite indicator: Each practice is a constituent component of the overall adoption level rather than a reflective manifestation of a single latent trait. Its validity therefore rests primarily on content validity—the practices were identified and validated through expert workshops aligned with recommended IPDM protocols for *M. roreri*, *P. palmivora*, and *C. foraseminis*—rather than on internal consistency criteria, which are not a prerequisite for formative indices.

Two groups of items were excluded before index construction. First, items capturing access to rural extension and technical assistance, membership in producer associations, participation in training, and access to experience exchanges and information materials were removed, because these dimensions are used as explanatory variables in the regression models; retaining them in the dependent variable would have introduced a mechanical overlap between the outcome and its predictors. Second, the two chemical control items were excluded on conceptual and empirical grounds: Chemical control is a last‐resort measure within an integrated, sustainability‐oriented framework, and these items showed near‐zero to negative corrected item–total correlations, indicating that chemical‐reliant farmers constitute a distinct behavioral profile rather than part of the same adoption gradient.

The final index comprised 21 practices in four dimensions: sanitation (13), plantation establishment and management (4), administration (2), and reproduction and genetic improvement (2).

For each farmer, the INAI was computed as the proportion of adopted practices over the full set, assigning equal weight to each practice. Equal weighting was adopted because no empirical evidence or theoretical framework justified assigning differential importance across the recommended IPDM practices. Moreover, all practices are considered complementary components of an integrated management strategy, and the omission of any individual practice may reduce the overall effectiveness of pest and disease management. Equal weighting has been widely applied in agricultural innovation adoption indices, particularly when objective information for deriving alternative weights is unavailable. To assess the robustness of this assumption, a sensitivity analysis based on dimension‐level aggregation was conducted, yielding highly consistent results with the original specification.

As a sensitivity analysis, a two‐level scheme averaging each dimension separately and then averaging the four dimension scores was also computed; the two schemes were almost perfectly correlated (*r* = 0.89) and yielded identical substantive conclusions. Internal consistency of the final set, assessed with the Kuder–Richardson 20 coefficient appropriate for dichotomous items, was acceptable (KR‐20 = 0.75), and all corrected item–total correlations were positive.

The resulting INAI was a continuous proportion lying strictly within the open unit interval, with no observations at either boundary. Beta regression was therefore applied directly, without boundary transformation, and fractional regression—which additionally admits boundary values—was estimated as a robustness check; both specifications yielded consistent estimates.

### 2.5. Econometric Model Specification

To identify the factors associated with innovation adoption in cacao pest and disease management, two complementary econometric approaches were employed: beta regression and fractional regression. Both models are appropriate for dependent variables expressed as proportions bounded between 0 and 1 and have been widely applied in studies of agricultural technology adoption and efficiency [44, 45].

The dependent variable was the INAI, defined as the proportion of recommended IPDM practices adopted by each farmer. Following the revision of the index construction procedure (Section 2.4), the final INAI ranged from 0.058 to 0.981, with no observations at either boundary value. Consequently, beta regression was considered appropriate for modeling the conditional mean of the index without requiring boundary transformations.

Under the beta regression framework, the INAI was assumed to follow a beta distribution parameterized by its conditional mean and a precision parameter, as shown in the following equation:

(2) Yi∼Beta μi, φ,

where *Y*_*i*_ denotes the INAI of farmer *i*, *μ*_*i*_ = *E* (*Y*_*i*_|*X*_*i*_) is the conditional mean of the index, and *φ* is the precision parameter.

Under this parameterization, the conditional variance depends on both the mean and the precision parameter according to the following equation:

(3) Var YiXi=μi 1−μi1+φ.

This formulation allows the variance to vary as a function of the mean, a common feature of proportional data [44]. As *μ*_*i*_ approaches either boundary of the unit interval, the variance decreases, whereas intermediate values are associated with greater variability.

The conditional mean was related to the explanatory variables through a logit link function, as specified in the following equation:

(4) logμi/1−μi=Xi′β,

where *X*_*i*_ is the vector of explanatory variables and *β* is the vector of parameters to be estimated. Parameters were estimated by maximum likelihood using the betareg package in R [46].

As a robustness analysis, a fractional regression model following Papke and Wooldridge [45] was also estimated. Although the revised INAI did not contain observations at 0 or 1, fractional regression was used as a complementary specification because it provides a quasi‐likelihood framework that does not require the dependent variable to follow a beta distribution and is less sensitive to distributional misspecification. The fractional model therefore served as a robustness check for the beta regression results.

Following Papke and Wooldridge [45], the conditional mean in the fractional model was specified according to the following equation:

(5) EYiXi=GXi′β,

where *G*(*·*) denotes the logistic cumulative distribution function, *X*_*i*_ is the vector of explanatory variables, and *β* is the vector of parameters to be estimated. The model was estimated using a generalized linear model with a logit link and quasi‐binomial variance specification [45, 47].

The explanatory variables included farmer characteristics (age, education, sex, and years of farming experience), household characteristics (household size), farm characteristics (total farm area, plantation age, and cacao yield), and institutional factors (access to agricultural credit, access to extension services, association membership, and participation in training activities). These variables were selected based on previous empirical studies of agricultural innovation adoption and rural advisory systems. Total cacao area was excluded from the final specification because it is nested within total farm area and did not improve model fit. Variance inflation factor (VIF) diagnostics indicated no evidence of problematic multicollinearity among the retained predictors (all VIF values < 2.3).

Because the coefficients in Equations (4) and (5) are expressed on the logit scale, they are not directly interpretable in terms of changes in the adoption index. Therefore, average marginal effects (AME) were calculated for all explanatory variables. AME quantify the average change in the predicted INAI associated with a one‐unit increase in a continuous variable or a change in category for binary variables, thereby facilitating interpretation of effect magnitudes on the original 0–1 scale.

Model adequacy was evaluated using pseudo‐*R* ^2^, residual diagnostics, and sensitivity analyses. The significance of model coefficients was assessed using Wald tests. Additional robustness checks included re‐estimation using municipality‐clustered standard errors and the exclusion of influential observations identified through Cook’s distance. The consistency of coefficient signs, statistical significance, and marginal effects across the beta and fractional specifications was used to assess the robustness of the estimated associations.

Differences in the INAI across municipalities were assessed with the Kruskal–Wallis test, a nonparametric alternative to one‐way ANOVA appropriate given the departure of the index from normality (Shapiro–Wilk, *p* < 0.01). All analyses were conducted in R using the betareg, glm, sandwich, lmtest, and marginaleffects packages [48].

## 3. Results and Discussion

### 3.1. INAI

The INAI for cacao pest and disease management averaged 0.63, indicating a moderate level of technology adoption among farmers. Adoption varied across the four practice categories (Figure 3), being highest for plantation establishment and management and lowest for administration.

**FIGURE 3:**
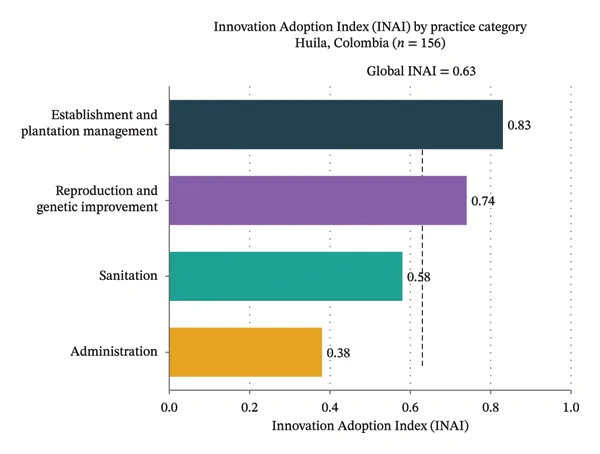
Innovation Adoption Index (INAI) by category for cacao disease management in Huila (Colombia).

The establishment and plantation management category showed the highest adoption (0.83). This is consistent with Jaimes‐Suárez et al. [40], who emphasize the importance of formation and maintenance pruning in optimizing plant development and disease prevention. Effective pruning improves plant structure and increases resilience to pathogens, such as *M. roreri* and *P. palmivora*.

The reproduction and genetic improvement category (0.74) reflected a relatively high level of adoption, suggesting a growing integration of genetic selection and improved planting materials, although further expansion of high‐yielding, disease‐resistant varieties remains possible. In contrast, sanitation (0.58) and, in particular, administration (0.38) exhibited the lowest adoption, identifying critical gaps in disease control and farm management. The sanitation score points to difficulties in implementing field hygiene and disease control practices, such as weekly sanitary rounds, removal of affected pods, and biological control, whereas the low administration score reflects limited record‐keeping and activity planning, which may constrain farmers’ capacity to plan and manage new technologies.

### 3.2. Socioeconomic and Demographic Profile of Smallholder Cacao Farmers in Huila, Colombia

Mean INAI values by municipality are reported in Table 2 for descriptive purposes. A Kruskal–Wallis test across all six municipalities was statistically significant (*H* = 15.16, *p* = 0.010); however, this result was driven by Hobo and Yaguará, surveyed with only six and four farmers, respectively. Restricting the comparison to the four municipalities with adequate samples (Gigante, Algeciras, Campoalegre, and Rivera; *n* ≥ 27) yielded no significant differences (*H* = 3.25, *p* = 0.355), with mean INAI ranging narrowly from 0.61 to 0.69. The apparent municipal variation therefore reflects the very small, likely nonrepresentative samples in Hobo and Yaguará rather than a robust geographical pattern, and results for these two municipalities should be interpreted descriptively and with caution.

**TABLE 2:** Socioeconomic and farm characteristics of cacao producers in Huila, Colombia.

Characteristic	Algeciras	Campoalegre	Gigante	Hobo	Rivera	Yaguará
*N*	40	38	41	6	27	4
INAI	0.69 ± 0.17	0.63 ± 0.20	0.61 ± 0.21	0.43 ± 0.15	0.65 ± 0.16	0.44 ± 0.03
Age (yr)	58.4 ± 12.5	58.8 ± 13.6	57.0 ± 14.2	57.5 ± 8.8	61.2 ± 13.7	51.8 ± 9.9
Education (yr)	6.9 ± 4.3	7.8 ± 4.9	8.4 ± 5.0	9.7 ± 2.4	11.4 ± 4.8	9.3 ± 2.1
Experience (yr)	15.3 ± 11.7	20.0 ± 14.4	23.2 ± 16.0	13.3 ± 5.3	26.4 ± 20.2	14.0 ± 4.7
Family size	3.5 ± 1.8	3.6 ± 1.9	3.2 ± 1.4	2.7 ± 0.8	2.9 ± 1.3	3.0 ± 0.8
Farm area (ha)	4.5 ± 3.1	4.9 ± 3.9	8.9 ± 11.3	2.4 ± 0.7	5.2 ± 11.2	1.5 ± 0.4
Cacao area (ha)	1.95 ± 0.98	2.26 ± 1.18	2.77 ± 2.58	2.25 ± 0.76	2.19 ± 1.98	1.13 ± 0.63
Plantation age (yr)	13.1 ± 7.4	21.3 ± 16.2	18.6 ± 15.8	13.2 ± 5.5	18.4 ± 12.5	12.0 ± 5.4
Yield (t ha^−1^)	0.61 ± 0.43	0.48 ± 0.33	0.62 ± 0.54	0.38 ± 0.16	0.50 ± 0.40	0.23 ± 0.05
Planting density (plants ha^−1^)	1064 ± 118	1084 ± 345	1084 ± 314	1111 ± 0	1211 ± 654	1111 ± 0

*Note:* INAI values reflect the revised 21‐practice index. Values are mean ± SD. Planting density was derived from the reported planting distances as 10,000/(*a* × *b*), in plants ha^−1^.

Among the well‐sampled municipalities, Algeciras recorded the highest mean INAI (0.69 ± 0.17) and Gigante the lowest (0.61 ± 0.21), but, as noted, these differences were not statistically significant. The socioeconomic and demographic characteristics of the surveyed farmers nonetheless illustrate the diversity of the cacao sector in Huila (Table 2).

The average age of farmers ranged from 51.75 years in Yaguará to 61.22 years in Rivera, reflecting an aging farming population. Mean schooling ranged from 6.93 ± 4.34 years in Algeciras to 11.44 ± 4.75 years in Rivera. Farming experience spanned from 13.33 years in Hobo to 26.44 years in Rivera, and mean household size from 2.67 in Hobo to 3.55 in Campoalegre.

Landholdings also varied: Gigante had the largest average farm area (8.89 ± 11.25 ha) and Yaguará the smallest (1.50 ± 0.41 ha), with the cacao area ranging from 1.13 ha in Yaguará to 2.77 ha in Gigante. Mean plantation age ranged from 12.00 years in Yaguará to 21.32 years in Campoalegre and cocoa yield ranged from 0.23 ± 0.05 t·ha^−1^ in Yaguará to 0.62 ± 0.54 t·ha^−1^ in Gigante. Possible links between these characteristics and adoption (e.g., schooling, experience, household labor, or farm size) are examined formally in the regression analysis (Section 3.5) and discussed below, rather than inferred from the descriptive comparison.

Planting density, derived from the reported planting distances, was relatively uniform across the sample: 80.8% of farms used a 3 × 3 m arrangement (≈1111 plants ha^−1^) and 87.2% fell within the conventional 3–4 m range, giving a mean density of approximately 1100 plants ha^−1^ (median 1111; range 278–4444 plants ha^−1^). Planting material consisted predominantly of interclonal hybrids and improved clones—such as CCN51, ICS95, FSV41, FEAR5, and San Vicente 41—frequently combined within the same plot.

### 3.3. Co‐Occurrence of Practices for Managing Cacao Pod Diseases and Borers

The joint adoption of practices was examined using the phi coefficient (the Pearson correlation between binary adoption indicators), which measures association beyond what would be expected from each practice’s individual prevalence. The full matrix for the 21 practices is shown in Figure 4, and the corresponding network of associations (phi ≥ 0.35) in Figure 5; in both, practices were ordered by hierarchical clustering using Ward’s linkage on a 1 − |phi| distance.

**FIGURE 4:**
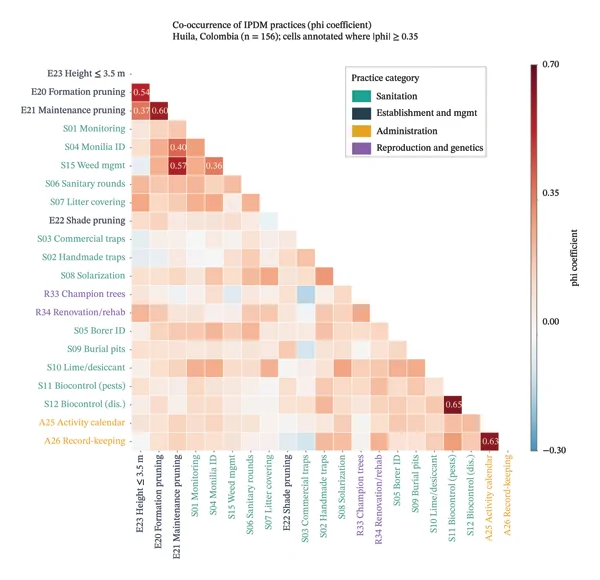
Co‐occurrence of integrated pest and disease management (IPDM) practices among smallholder cacao farmers in Huila, Colombia (*n* = 156), measured as the phi coefficient (Pearson’s correlation between binary adoption indicators) for the 21 practices retained in the adoption index. Only the lower triangle is shown; cells are annotated where |phi| ≥ 0.35.

**FIGURE 5:**
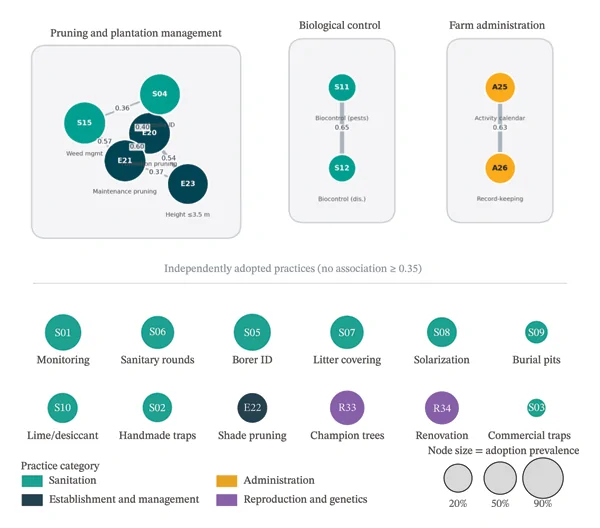
Co‐occurrence network of IPDM practices in Huila, Colombia (*n* = 156). Nodes represent practices, with color indicating the practice category and size proportional to adoption prevalence; edges connect pairs with phi ≥ 0.35, with edge width proportional to the strength of association and phi values shown on each edge. Three association bundles emerge—pruning and plantation management, biological control, and farm administration—whereas the remaining practices show no association ≥ 0.35 and are displayed separately as independently adopted.

Most pairs of practices were only weakly associated: The large majority of phi values fell below 0.20 (Figure 4), indicating that practices were generally adopted independently of one another. Only a small number of pairs were strongly associated, and these formed three coherent bundles (Figure 5).

The first bundle grouped pruning and plantation management practices. Formation and maintenance pruning co‐occurred strongly (phi = 0.60), and both were associated with maintaining trees below 3.5 m (phi = 0.54 and 0.37, respectively) and with weed management (phi = 0.57 for maintenance pruning). Monilia identification was also linked to this group, although more weakly (phi = 0.36–0.40). These practices were among the most widely adopted in the sample, with adoption prevalences above 0.90 for several of them.

The second bundle comprised the two biological control practices: biological control of pests and of diseases which showed the strongest association in the entire matrix (phi = 0.65). Notably, both had low overall adoption (approximately 0.25–0.27), indicating that, although few farmers used biological control, those who did tended to apply it against both pests and diseases. The third bundle linked the two administrative practices of keeping an activity calendar and record‐keeping (phi = 0.63) which were likewise adopted by a minority of farmers (approximately 0.38).

The remaining practices—including pest monitoring, handmade and commercial traps, sanitary rounds, litter covering, solarization, burial pits, lime or desiccant application, shade pruning, champion‐tree identification, and plantation renovation—showed no association ≥ 0.35 with any other practice and are displayed separately in Figure 5 as independently adopted. Among these, commercial traps stood out for their very low adoption (0.038), whereas others, such as pest monitoring and champion‐tree identification, were widely adopted (approximately 0.79–0.84) yet were not statistically associated with any specific companion practice.

Overall, and apart from the three internally consistent bundles (pruning and plantation management, biological control, and administration), IPDM practices were largely adopted in isolation rather than as integrated packages (Figures 4 and 5).

### 3.4. Social Networks and Cooperation

The technical knowledge exchange network comprised 218 nodes—156 farmers, 39 extension agents, and 23 institutional actors—connected by approximately 320 directed ties. The network was sparse (graph density = 0.007; mean degree = 2.9) and fragmented into one large connected component and several small peripheral ones. Reciprocity was very low (0.05), indicating that ties overwhelmingly reflected directed consultation—farmers seeking information—rather than mutual exchange (Figure 6).

**FIGURE 6:**
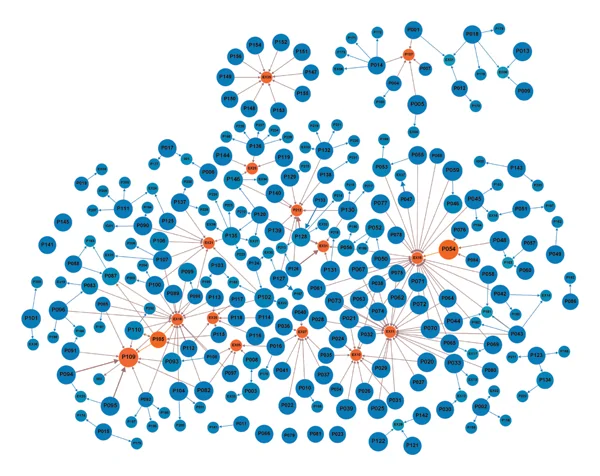
Technical knowledge network among cacao farmers in Huila. The circle size represents the Innovation Adoption Index (INAI) value, with larger nodes corresponding to farmers (P) who exhibit a higher level of innovation adoption. Orange‐colored nodes indicate actors with a higher in‐degree, meaning they are frequently consulted within the network. The network includes farmers (P), extension agents (EX), government institutions (IG), and research or educational institutions (II), illustrating the structure of knowledge exchange in the region.

In‐degree was highly concentrated: A small set of actors, predominantly extension agents (notably EX15, EX11, EX18, EX21, EX35, and EX26), received a disproportionate share of incoming ties, producing pronounced in‐degree centralization, whereas out‐degree was far more evenly distributed. Acting as bridges between scientific knowledge and field‐level practice, these agents are frequently consulted for crop‐management guidance and are central to disseminating technical recommendations and best practices among producers.

Direct farmer‐to‐farmer ties were comparatively scarce. Nevertheless, several of the highest‐adopting farmers (the largest nodes in Figure 6; e.g., P109, P054, and P105) were themselves among the most frequently consulted actors, suggesting that they can function as local knowledge brokers. Because farmer‐level network position is plausibly both a determinant and a consequence of adoption, and because the network and the INAI were drawn from the same cross‐sectional survey, centrality measures were treated as descriptive and were not entered as predictors in the beta and fractional regression models, so as to avoid simultaneity and reverse causality bias. The network evidence is therefore complementary to, rather than a formal test of, the adoption models, and integrating farmer network position into adoption models is a priority for future longitudinal research.

This centralized, weakly reciprocal structure has clear implications for diffusion. The scarcity of horizontal ties—particularly among high‐INAI farmers such as P109 and P054, who could serve as local innovation hubs—represents an underused channel for peer learning. De Roo et al. [49] emphasize that farmer‐driven knowledge exchange is key to reinforcing sustainable practices and promoting adoption, and the present pattern aligns with evidence from Indonesia, where social influence in centralized networks spreads practices largely through well‐connected farmers acting as information hubs [50]. Likewise, García‐Sánchez et al. [51] stress that strong farmer–extensionist relationships not only facilitate technical transfer but also build the trust and social cohesion needed to sustain long‐term innovation.

The network structure suggests that leveraging the social influence of high‐INAI farmers, while strengthening horizontal farmer‐to‐farmer interactions, could improve technology diffusion and adoption. Although extension agents remain central to knowledge dissemination, fostering peer‐led training sessions, participatory learning groups, and farmer‐to‐farmer mentoring could strengthen social learning and raise adoption rates.

### 3.5. Determinants of Innovation Adoption for Cacao Pod Disease and Borer Management: Beta and Fractional Regression Models

Beta and fractional regression models were estimated with the INAI as the dependent variable to identify the factors associated with the adoption of IPDM innovations. The two approaches yielded highly consistent results in the sign, magnitude, and statistical significance of the coefficients (Table 3).

**TABLE 3:** Determinants of innovation adoption (INAI) in cacao pest and disease management: Beta and fractional regression estimates.

Variable	Beta model		Fractional model	
	Coef. (SE)	*p*	Coef. (SE)	*p*
Intercept	−0.563 (0.496)	0.256	−0.741 (0.526)	0.161
Age (years)	−0.002 (0.005)	0.657	−0.001 (0.006)	0.871
Education (years)	0.018 (0.014)	0.212	0.019 (0.015)	0.202
Sex (male)	−0.166 (0.123)	0.178	−0.133 (0.131)	0.312
Years of experience	−0.004 (0.004)	0.400	−0.004 (0.005)	0.329
Household size	0.015 (0.038)	0.696	0.021 (0.040)	0.608
Total farm area (ha)	−0.022 (0.008)^∗∗^	0.004	−0.022 (0.008)^∗∗^	0.009
Plantation age (years)	−0.001 (0.005)	0.790	0.000 (0.005)	0.961
Yield (t ha‐1)	0.353 (0.147)^∗^	0.016	0.351 (0.158)^∗^	0.028
Credit use (yes)	0.374 (0.143)^∗∗^	0.009	0.328 (0.152)^∗^	0.033
Extension service (yes)	0.497 (0.170)^∗∗^	0.003	0.456 (0.179)^∗^	0.012
Association membership (yes)	0.104 (0.167)	0.534	0.190 (0.176)	0.282
Training participation (yes)	0.627 (0.272)^∗^	0.021	0.629 (0.286)^∗^	0.029
*φ* (precision)	6.864 (0.735)^∗∗∗^	< 0.001	—	
Dispersion (fractional)	—		0.137	
Pseudo‐*R* ^2^/residual dev.	0.233		20.68 (143 df)	
Observations	156		156	

*Note:* Coefficients on the logit scale; standard errors in parentheses. Reference categories: female (sex); no (credit, extension, association, training). Total cacao area was excluded for parsimony as it is nested within total farm area and nonsignificant; variance inflation factors confirmed no collinearity (all VIF < 2.3). *φ* is the beta precision parameter; the fractional model is estimated by quasi‐likelihood [45].

^∗^ *p* < 0.05, ^∗∗^ *p* < 0.01, ^∗∗∗^ *p* < 0.001.

Among farm‐level variables, total farm area was negatively associated with the INAI in both specifications (*β* = −0.022, *p* = 0.004 in the beta model; *β* = −0.022, *p* = 0.009 in the fractional model), whereas cacao yield was positively associated with adoption (*β* = 0.353, *p* = 0.016; and *β* = 0.351, *p* = 0.028, respectively).

Among institutional and support‐related variables, credit use, access to extension services, and participation in training were positively and significantly associated with the INAI in both models. In the beta specification, the coefficients were 0.374 (*p* = 0.009) for credit use, 0.497 (*p* = 0.003) for extension services, and 0.627 (*p* = 0.021) for training participation, with comparable estimates in the fractional model (Table 3). Association membership was not significantly associated with the INAI in either specification (*p* > 0.05).

The AME, reported in Table 4 to aid interpretation, indicated that training participation was associated with an average increase of 0.148 points in the predicted INAI (95% CI: 0.019–0.276; *p* = 0.024) and access to extension services with an increase of 0.116 points (95% CI: 0.036–0.195; *p* = 0.004) in the beta model. Credit use was associated with an increase of 0.082 points (95% CI: 0.022–0.141; *p* = 0.007). Among continuous variables, total farm area showed a negative association (AME = −0.005; 95% CI: −0.008 to −0.002; *p* = 0.004) and yield a positive one (AME = 0.079; 95% CI: 0.015–0.142; *p* = 0.015). The fractional model produced AME of similar magnitude and direction. No significant associations were detected for age, education, sex, years of experience, household size, plantation age, or association membership.

**TABLE 4:** Average marginal effects (AME) on the predicted INAI.

Variable	Beta model		Fractional model	
	AME (95% CI)	*p*	AME (95% CI)	*p*
Age (years)	−0.001 (−0.003, 0.002)	0.657	0.000 (−0.003, 0.002)	0.871
Education (years)	0.004 (−0.002, 0.010)	0.212	0.004 (−0.002, 0.011)	0.198
Sex (male vs female)	−0.037 (−0.090, 0.017)	0.176	−0.030 (−0.087, 0.027)	0.309
Years of experience	−0.001 (−0.003, 0.001)	0.399	−0.001 (−0.003, 0.001)	0.326
Household size	0.003 (−0.002, 0.010)	0.696	0.005 (−0.013, 0.022)	0.607
Total farm area (ha)	−0.005 (−0.008, −0.002)^∗∗^	0.004	−0.005 (−0.008, −0.001)^∗∗^	0.007
Plantation age (years)	0.000 (−0.002, 0.002)	0.790	0.000 (−0.002, 0.002)	0.960
Yield (t ha^−1^)	0.079 (0.015, 0.142)^∗^	0.015	0.079 (0.010, 0.148)^∗^	0.025
Credit use (yes vs no)	0.082 (0.022, 0.141)^∗∗^	0.007	0.072 (0.008, 0.136)^∗^	0.027
Extension service (yes vs no)	0.116 (0.036, 0.195)^∗∗^	0.004	0.106 (0.023, 0.190)^∗^	0.013
Association membership (yes vs no)	0.023 (−0.051, 0.098)	0.537	0.043 (−0.036, 0.123)	0.287
Training participation (yes vs no)	0.148 (0.019, 0.276)^∗^	0.024	0.148 (0.013, 0.284)^∗^	0.032

*Note:* AME express the average change in the predicted INAI (0–1) for a one‐unit increase (continuous predictors) or a category change (binary predictors); 95% confidence intervals in parentheses. AME replace odds ratios, which are not interpretable for a continuous proportional outcome.

^∗^ *p* < 0.05, ^∗∗^ *p* < 0.01.

Robustness analyses are summarized in Table 5. Under municipality‐clustered standard errors and after re‐estimating the model excluding eight influential observations (Cook’s *D* > 4/*n*), the positive associations of extension services, training participation, and credit use, and the negative association of total farm area, remained statistically significant. Yield remained significant after excluding influential observations but was only marginally significant under clustered standard errors (*p* = 0.071); association membership was nonsignificant throughout. Considered jointly, the three institutional variables (extension services, association membership, and training participation) significantly improved model fit (likelihood‐ratio test: *χ* ^2^ = 16.56, df = 3, *p* < 0.001).

**TABLE 5:** Robustness of the main effects (beta model): *p* values under conventional standard errors, municipality‐clustered standard errors, and after excluding eight influential observations (Cook’s *D* > 4/*n*).

Predictor	Conventional SE	Cluster‐robust SE	Excl. influential
Extension service	0.003^∗∗^	< 0.001^∗∗∗^	0.012^∗^
Training participation	0.021^∗^	< 0.001^∗∗∗^	0.004^∗∗^
Credit use	0.009^∗∗^	0.009^∗∗^	0.001^∗∗^
Total farm area	0.004^∗∗^	< 0.001^∗∗∗^	0.026^∗^
Yield	0.016^∗^	0.071	< 0.001^∗∗∗^
Association membership	0.534	0.355	0.785

*Note:* The institutional block (extension + association + training) was jointly significant in all specifications (likelihood‐ratio test: *χ* ^2^ = 16.56, df = 3, *p* < 0.001). Yield was significant under conventional errors but only marginal under clustered errors, indicating limited robustness. ^∗^ *p* < 0.05, ^∗∗^ *p*  < 0.01, ^∗∗∗^ *p* < 0.001.

Training participation and access to extension services showed the most consistent and sizeable associations with innovation adoption for cacao pest and disease management, remaining significant across both regression specifications and ranking among the largest marginal effects in the study. Training participation was associated with an increase of roughly 0.15 points in the predicted INAI and extension access with roughly 0.11 points. These patterns are consistent with prior work emphasizing the role of technical assistance, farmer learning, and advisory services in agricultural innovation adoption [52–54]. Because the data are cross‐sectional and access to these services is not randomly assigned, however, the associations cannot be interpreted causally (see limitations below).

The magnitude of the training association is particularly noteworthy. Participatory learning approaches, such as farmer field schools, demonstrations, and group training, facilitate the acquisition of technical knowledge and strengthen farmers’ capacity to implement recommended practices [55]. In IPDM, where effective management typically requires the coordinated implementation of several practices, access to training may be especially important for translating technical recommendations into on‐farm decisions.

Access to credit was also positively associated with the INAI, with farmers using credit showing adoption levels approximately 0.07–0.08 points higher than nonusers. This is consistent with evidence that financial resources ease the liquidity constraints and investments associated with adoption [56, 57]. The credit association was nonetheless smaller than those of training and extension, suggesting that knowledge‐related factors may play a more prominent role than financial access in explaining adoption within this population.

Farm size was negatively associated with adoption across specifications. Although larger farms are often expected to benefit from economies of scale, comparable negative relationships have been reported in agroforestry and cacao systems where management complexity rises with area [58]. The modest per‐hectare marginal effect may reflect differences in management intensity, labor allocation, or the logistical difficulty of implementing multiple IPDM practices over larger areas; these mechanisms would need to be evaluated directly in future work.

Yield was positively associated with adoption in both models, but this relationship was only marginally significant under municipality‐clustered standard errors and should therefore be interpreted with particular caution. Beyond its limited statistical robustness, yield is plausibly an outcome of adoption rather than a driver of it: More intensive management may simultaneously raise productivity and adoption levels. This potential reverse causality, together with the cross‐sectional design, means the yield association should not be read directionally.

Association membership was not significantly associated with the INAI once institutional‐support items had been removed from index construction and training and extension access were controlled for. This suggests that the relationship commonly observed between farmer organizations and adoption may operate indirectly—through expanded access to training, technical assistance, and knowledge exchange—rather than through membership per se. Consistent with this interpretation, the institutional variables were jointly significant even though association membership was not individually so. Farmer organizations thus remain important actors in the innovation system, but membership alone was not associated with higher adoption once these pathways were explicitly accounted for.

These findings should be interpreted in light of two limitations. First, because access to extension, training, and credit is not randomly assigned, the estimated associations may partly reflect self‐selection of more proactive or better‐resourced farmers into these services; establishing causal effects would require longitudinal or quasi‐experimental designs. Second, the models explained a modest share of the variation in adoption (pseudo‐*R* ^2^ *= *0.23), indicating that unobserved factors—such as farmer motivation, risk preferences, or local agroecological conditions—also shape adoption decisions.

Finally, the SNA complements the regression results by showing that extension agents and institutional actors occupied central positions in the knowledge exchange network, consistent with studies underscoring the role of information networks in the diffusion of management practices [24, 59]. Although farmer‐level network indicators were not incorporated into the econometric models, the network structure offers additional, complementary evidence that access to information and learning opportunities may be important pathways for innovation diffusion among cacao producers. Integrating farmer‐level network position (e.g., degree centrality) directly into adoption models is a promising direction for future research.

## 4. Conclusion

This study found that, among smallholder cacao farmers in Huila, higher adoption of sustainable pest and disease management practices was most consistently associated with participation in training, access to extension services, and the use of credit, whereas total farm area was negatively associated with adoption. Although association membership was not individually associated with adoption once these channels were accounted for, the institutional block as a whole was jointly significant, suggesting that producer organizations may influence adoption indirectly—by broadening access to training, technical assistance, and knowledge exchange—rather than through membership itself. The low adoption of several practices, notably commercial traps, biological control, and farm record‐keeping, points to persistent gaps that targeted technical assistance could help close.

Social networks play an important role in technology diffusion: Extension agents occupied central positions as knowledge brokers, whereas direct farmer‐to‐farmer ties were comparatively scarce. This centralized, weakly reciprocal structure suggests that peer‐learning mechanisms—such as farmer field schools, participatory learning groups, and farmer‐to‐farmer mentoring—remain underused. Overall, the findings indicate that an integrated approach combining technical training, financial access, and stronger horizontal farmer networks is likely to be more effective than any single instrument for closing adoption gaps and supporting the sustainability of the cacao sector.

Several limitations qualify these conclusions and define priorities for future work. Because the data are cross‐sectional and access to training, extension, and credit is not randomly assigned, the estimated relationships are associations rather than causal effects; longitudinal panel data and quasi‐experimental designs—for example, difference‐in‐differences or instrumental‐variable approaches that exploit the staggered rollout of extension and credit programs—would be needed to identify causal effects and to address self‐selection into these services. The models also explained a modest share of the variation in adoption, indicating that unobserved factors, such as farmer motivation, risk perception, and market access, should be incorporated in future studies, ideally alongside shade‐canopy composition, which was not recorded here. Finally, because the network indicators were descriptive, future research should integrate farmer‐level network position (e.g., degree centrality) directly into adoption models, quantify information flows, and extend the analysis to other cacao‐producing regions to assess the generalizability of these findings beyond Huila.

## Author Contributions

Sonia Mercedes Polo‐Murcia: writing–review and editing, writing–original draft, visualization, validation, supervision, methodology, investigation, formal analysis, data curation, and conceptualization. Diego Alberto Navarro‐Niño: investigation, writing–original draft, validation, and conceptualization. César Augusto Terán‐Chaves: writing–review and editing, writing–original draft, visualization, validation, supervision, software, methodology, investigation, formal analysis, data curation, and conceptualization. Eleonora Rodríguez‐Polanco: writing–review and editing and funding acquisition. Bayardo Parra‐Alferes: writing–original draft. Jhon Jairo Zuluaga‐Peláez: writing–original draft.

## Funding

This research was funded by the General System of Royalties (SGR) through the project “Development and validation of integrated crop and agroindustry management technologies to enhance the competitiveness and sustainability of the cacao production system in Campoalegre, Gigante, Rivera, and Algeciras (Huila, Colombia)” (BPIN Code 2021000100166).

## Ethics Statement

This study was conducted in accordance with the relevant ethical standards.

## Consent

Informed consent was obtained from all individual participants included in the study. All authors have consented to the publication of the manuscript.

## Conflicts of Interest

The authors declare no conflicts of interest.

## Data Availability

The data that support the findings of this study are available from the corresponding author upon reasonable request.
